# Recognition of Bimolecular Logic Operation Pattern Based on a Solid-State Nanopore

**DOI:** 10.3390/s21010033

**Published:** 2020-12-23

**Authors:** Han Yan, Zhen Zhang, Ting Weng, Libo Zhu, Pang Zhang, Deqiang Wang, Quanjun Liu

**Affiliations:** 1 State Key Laboratory of Bioelectronics, School of Biological Science and Medical Engineering, Southeast University, No. 2, Sipailou, Nanjing 210096, China; 230209118@seu.edu.cn (H.Y.); 220181842@seu.edu.cn (Z.Z.); 230169449@seu.edu.cn (L.Z.); 2 Chongqing Institute of Green and Intelligent Technology, Chinese Academy of Sciences, Chongqing 400714, China; wengting@cigit.ac.cn (T.W.); zhangpang@cigit.ac.cn (P.Z.); dqwang@cigit.ac.cn (D.W.)

**Keywords:** nanopore, DNA logic gate, DNA tetrahedron, probe, AND operation

## Abstract

Nanopores have a unique advantage for detecting biomolecules in a label-free fashion, such as DNA that can be synthesized into specific structures to perform computations. This method has been considered for the detection of diseased molecules. Here, we propose a novel marker molecule detection method based on DNA logic gate by deciphering a variable DNA tetrahedron structure using a nanopore. We designed two types of probes containing a tetrahedron and a single-strand DNA tail which paired with different parts of the target molecule. In the presence of the target, the two probes formed a double tetrahedron structure. As translocation of the single and the double tetrahedron structures under bias voltage produced different blockage signals, the events could be assigned into four different operations, i.e., (0, 0), (0, 1), (1, 0), (1, 1), according to the predefined structure by logic gate. The pattern signal produced by the AND operation is obviously different from the signal of the other three operations. This pattern recognition method has been differentiated from simple detection methods based on DNA self-assembly and nanopore technologies.

## 1. Introduction

Since the advent of DNA computation, it has been receiving more and more attention due to its good biocompatibility. In a traditional electronic computer, a microprocessor uses basic logic gates (AND, OR, NOT, etc.) to construct an electronic circuit capable of performing Boolean mathematical logic. This is crucial for modern computers, and these computer concepts have a similar understanding in biology. In fact, a human body is likely a complex biological computer, which performs a lot of logical operations. A variety of materials have been used to construct logic gates such as enzymes, DNA, RNA, and other biological molecules [1,2,3,4,5,6].

Recently, DNA molecular hybridization processes and ribozyme-based logic operations have been widely considered to be logic gates. The design of a DNA logic gate mainly depends on the properties of DNA molecules, such as base pairing [7,8], single strand replacement [9], DNAzyme cleavage [10,11], nucleic acid aptamer binding, i-motif structure interconversion, and DNA tweezers [12,13,14]. In the design of DNA computing, biomolecules contain biological information, such as molecular spatial conformation, enzyme activity, catalytic properties, and recognition sites of enzymes, which can be converted into logic operations [15,16,17,18,19,20]. Over the past few years, this method has been often applied to detect biomarkers of various diseases. However, most of the decoding of the output terminal is still realized by PCR fluorescence amplification or gel electrophoresis [21,22], which are time-consuming.

In recent years, nanopores have been broadly developed for detecting and identifying biomolecules. In a typical setup, an external voltage drives a biomolecule through a nanometer-size pore in a thin supporting membrane, causing a characteristic temporary change in the translocation ionic current. Nanopore technology can be applied to assay the spatial structure [23,24,25], dynamic changes [25,26,27,28,29], and biochemical characteristics [30,31]. It is not necessary to carry out chemical modification, surface adsorption, and calibration object insertion, which may affect the activity of the sample. Due to its advantages of rapid reading and label-free detection [32,33,34,35], the nanopore platform has been developed into a powerful tool for detecting biomolecules. It has been applied to detect and analyze the types and structures of biomolecules, such as polymer morphology, morphology and sequencing of DNA and RNA [36], virus and antibody-virus interactions [37], morphology of amino acid [38], peptides and proteins [39,40,41,42,43,44,45,46], ribosomes, and chemical bond formation [47,48,49]. In addition, some synthetic nanostructures with special configurations have also been detected by nanopores, such as DNA tetrahedrons and other DNA origami structures [50,51,52,53]. In view of this, Ryuji Kawano et al. used biological nanopores to design a DNA logic gate for the detection of lung cancer markers [54]. Ulrich F. Keyser designed a DNA hard drive using glass nanopores, for storage, calculation, and reading of data in a variable DNA three-dimensional structure [55]. Inspired by these works, we have designed a kind of DNA logic gate based on a solid-state nanopore platform and DNA tetrahedrons. DNA tetrahedrons are highly rigid and well-defined structures with atomic precision and versatile functionality, and they provide scaffolds for anchoring of a variety of biomolecular probes (DNA, aptamers, peptides, and proteins) for biosensing. Significantly, this DNA nanostructure-based biosensing platform greatly increases target accessibility and improves the sensitivity for various types of molecular targets (DNA, RNA, proteins, and small molecules) by several orders of magnitude. In addition, the DNA tetrahedron also has the characteristics of a stable structure and uniform size. In recent years, several DNA-based sensors have been published with high sensitivity towards biomarker detection [56,57,58]. In order to realize the decoding application of a solid-state nanopore, it is necessary to obtain signals with a higher signal-to-noise ratio. To this end, we have constructed a logic operator assisted with a tetrahedral structure which can provide an improved signal-to-noise ratio and temporal resolution, due to its unique spatial structure and carried charges, as the DNA tetrahedron has been applied to build various biosensors [59,60,61,62,63,64,65,66].

**Figure 1:**
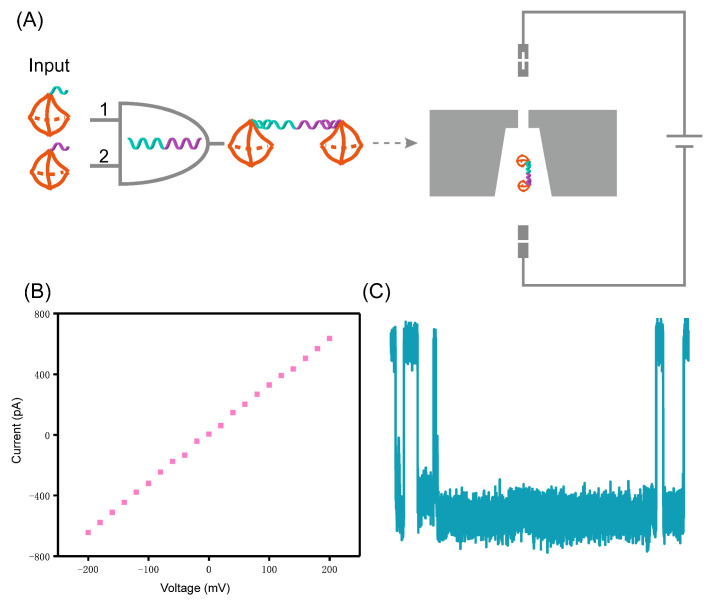
(**A**) Schematic illustration of the AND gate for marker molecules of lung cancer. When Probe 1 and Probe 2 are provided as inputs at the same time, they form a double tetrahedral structure with the marker molecules, causing a long blockage when translocated through the solid-state nanopore under an external voltage; (**B**) I-V characterization curve of the SiNx nanopore with a diameter about 3.5 nm in the range ±200 mV; (**C**) The signals after the AND operation.

In this paper, we designed a DNA logic gate operation based on nanopore measurement and pattern recognition of DNA computing technology. We attempted to construct an AND operation using a lung cancer marker recognition locus (miR-21), which included four operation patterns, i.e., (0, 0), (0, 1), (1, 0), (1, 1). The lung cancer marker recognition locus used in our experiments was converted into corresponding DNA. According to our experimental design, when two tetrahedron probes were presented at the same time, they formed a double tetrahedron structure with the marker molecule and resulted in a long blocking signal, when the structure translocated through the nanopore, as shown in the schematic diagram of Figure 1. By the analysis of duration and amplitude of the signal, the signal of (1, 1) can be distinguished from the other three patterns ((0, 0), (0, 1), (1, 0)).

## 2. Materials and Methods

### 2.1. Chemical and Instrument

All aqueous solutions were prepared using ultrapure water from a Milli-Q water purification system (Millipore Corporation, Billerica, MA, USA) and were filtered with 0.22 μm needle filter unit. Lithium chloride (LiCl) was purchased from Sigma. TE buffer (pH = 8) was composed of 10 mM Tris and 1 mM EDTA (ethylenediaminetetraacetic acid), of which Tris and EDTA were provided by Sigma. The electrolyte used in the experiment was a mixture of 1 M LiCl, 10 mM Tris and 1 mM EDTA. TM (Tris MgCl_2_) buffer (20 mM Tris, 50 mM MgCl_2_, pH = 8) was used in the synthesis of the tetrahedron probe. All the prepared solutions were stored in the refrigerator at −4 °C. The silicon nitride chips used in the experiments were purchased from Norcada. During the experiments, the signals were acquired through the patch clamp system (Axon Axopatch 200B, Molecular Devices, San Jose, CA, USA).

### 2.2. Nanopore Fabrication and Measurement

The size of nanopore chips are 3 × 3 mm^2^, showing a sandwich structure, in which the thickness of the upper and lower layers of silicon nitride is 15 nm. Firstly, the nanopore chip was mounted in the flow cell (Figure S1), and two Viton O-rings were used to seal the chambers to ensure that the nanopore was the only channel connecting the two chambers (cis and trans chamber). Then, the chamber was washed with ultrapure water and absolute ethanol three times alternately to increase the wettability of the nanopore sheet to avoid the generation of air bubbles during drilling and experiments. Finally, the two chambers were filled with electrolyte, and a 3.5 nm nanopore was fabricated on the Si_3_N_4_ freestanding membrane by dielectric breakdown [67]. After the nanopore was fabricated, it was also washed three times with ultrapure water and absolute ethanol alternately to ensure that the nanopore was clean. The open-pore current was tested by I-V cures before the experiment to calculate the diameter of the nanopore. This result is shown in Figure 1B.

### 2.3. Synthesis of Tetrahedron Probes

Each of the single stranded and the tetrahedron probes was dissolved in TM buffer, and then mixed in equal volume. The mixture was heated to 95 °C for 10 min in the water bath, and then quickly cooled to 4 °C. The prepared sample was stored in the refrigerator at −20 °C. For the logic operations, input DNAs (sequences listed in Table S1) were added in the mixture, and then incubated for 30 min to operate the logic gates under room temperature. In our experiment, each edge length of the synthesized DNA tetrahedral probe was 2.4 nm. The four oligonucleotide strands used to synthesize the tetrahedron probe and the DNA strands used in the experiment are displayed in the supporting information (Table S1). Gel electropherograms of different DNA configurations are displayed in the Supporting Information (Figure S2). The synthesis and characterization of tetrahedrons can be referred to in our previous work in detail [52].

## 3. Results and Discussion

**Figure 2:**
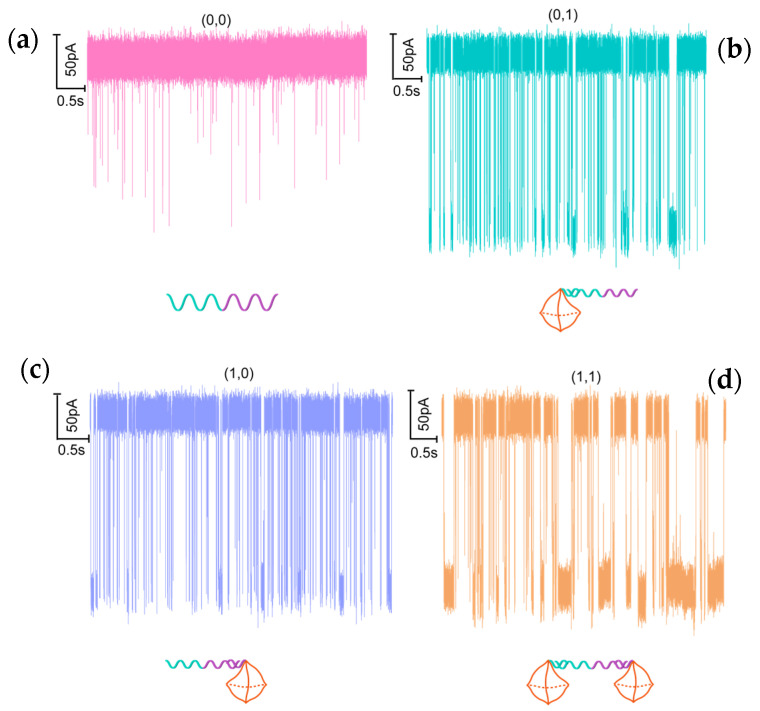
(**a**–**d**) Continuous current traces for different DNA logic operations (0, 0), (0, 1), (1, 0), (1, 1) translocation through a 3.5 nm diameter solid-state nanopore under 100 mV voltage in 1 M LiCl. The concentrations of tetrahedron Probe 1 and Probe 2 were 10 nM, respectively. The molar ratios of Probe 1/Probe 2/DNA(miR-21) are 0:0:1 in (0, 0), 0:1:1 in (0, 1), 1:0:1 in (1, 0), and 1:1:1 in (1, 1).

The nanopore can be used to detect the output biomolecules of DNA logic operations quickly in a label-free fashion. Before the logic operation experiments, we detected the two tetrahedron probes. Figure S3 shows the current traces from two tetrahedron probes (T_1_ and T_2_). The T_1_ tetrahedral probe is composed of A7, B7-C1, C7, and D7, while the T_2_ tetrahedral probe is composed of A7, B7-C2, C7, and D7. The analysis of amplitude and duration are shown in the Supporting Information (Figure S4). As shown in Figure S4, the average translocation amplitude and duration of T1 and T2 are 186.34 ± 0.301 pA, 6.35 ± 0.058 ms, and 186.82 ± 0.306 pA, 8.96 ± 0.140 ms, respectively. The results indicate that there is no significant difference in the amplitude and duration of the two probes, due to their similar structure. However, as compared with single-strand DNA (105.32 ± 0.287 pA and 0.15 ± 0.002 ms), the three-dimensional (3D) structure of DNA probes greatly increases the duration and amplitude. The main reason for this phenomenon is that the 3D spatial structure of the tetrahedral probe intensifies the interaction with the nanopore wall when passing through the nanopore. Figure S5 presents the duration and amplitude of the tetrahedron probes (T_1_ and T_2_) and corresponding complex of DNA-tetrahedron probe (Figure 2b (0, 1) and Figure 2c (1, 0)). As a result of the analysis, the amplitude and duration in the logical operation pattern of (0, 1) and (1, 0) can be clearly distinguished from T1 and T2. The tetrahedral probes with a longer chain length after the logic gate operation caused a larger amplitude and duration when translocating through the nanopore. Furthermore, we detected four operations, i.e., (0, 0), (0, 1), (1, 0), (1, 1) by the solid-state nanopore. Unless otherwise specified, the electrolyte used in the experiments is 1 M LiCl, the applied voltage is 100 mV, and the concentration of the detected analytes is 10 nM. Figure 2 shows the current traces for four operations. For (0, 0) it represents the DNA fragment (Table S1) corresponding to the lung cancer marker (miR-21), which only causes spike-like and short-lived translocation signals (Figure 2a). In the case of (0, 1) and (1, 0), as demonstrated in Figure 2b,c, they represent a complex of DNA and different molecular probes. As compared with the signals produced by single-stranded DNA, the complex of a single tetrahedral probe and the marker has a 3D spatial structure. When it translocates through the nanopore under the action of an external voltage, it interacts more strongly with the wall of the nanopore, resulting in a significant increase in translocation time (Figure 2b,c). For AND logic (1, 1), two input tetrahedron probes (Probe 1, and Probe 2) are designed to combine into the double tetrahedron structures (Figure 2d). As a result, we can observe the long-lived translocation blockage signals (output = 1) only when both inputs are present (input = (1, 1)); otherwise (input = (0, 0), (1, 0), or (0, 1)) the duration level is low (output = 0). It can be found intuitively from the recorded current curves. From the current traces, we can also intuitively observe that the proportion of long-lived translocation signals has increased significantly after the AND operation.

**Figure 3:**
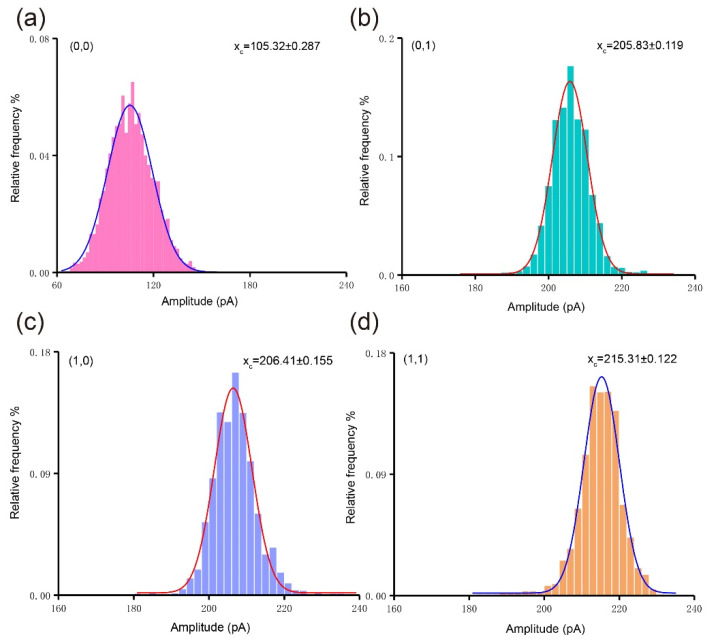
(**a**–**d**) Histograms of the amplitude for each AND operation pattern. Solid lines are Gaussian fitted curves for each group of data.

Furthermore, the data from four DNA logic operations are analyzed. The mean amplitude of (0, 0), (0, 1), (1, 0), (1, 1) was 106.17 ± 0.95 pA, 205.82 ± 0.78 pA, 206.79 ± 0.99 pA, and 215.28 ± 0.55 pA, respectively, as shown in Figure 3. According to the results of the analysis, the presence of the tetrahedral probe increases the translocation amplitude and also increases the signal-to-noise ratio. It can be found that the amplitude of (1, 1) is slightly different from the amplitude of (0, 1) and (1, 0), however the amplitude of (0, 1) is almost the same as that of (1, 0). It is reasonable that the configuration is almost the same for Probe 1 and Probe 2 (T1 tetrahedral probe is composed of A7, B7-C1, C7, and D7, while T2 tetrahedral probe is composed of A7, B7-C2, C7, and D7), the same tetrahedron with a single strand that is partially complementary to the DNA fragment (miR-21). The slight difference in amplitude between (0, 1), (1, 0) and (1, 1) might be caused by the almost uniform lateral occupation ratio of the molecular translocation through the nanopore. It means that the double tetrahedron structure has no advantage over the single tetrahedron in increasing the signal amplitude. Therefore, based on the analysis of amplitude alone, we cannot distinguish the three patterns of logic operation ((0, 1), (1, 0), and (1, 1)).

**Figure 4:**
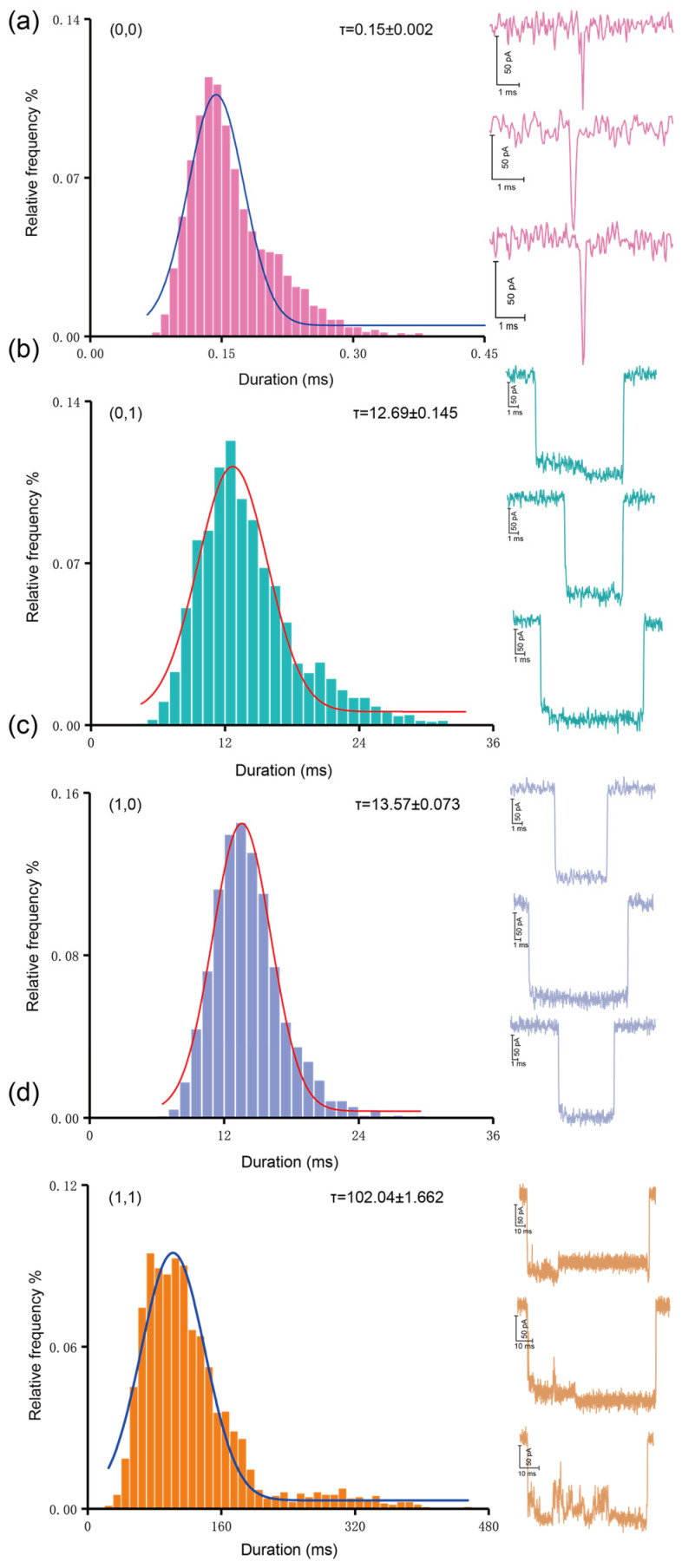
(**a**–**d**) Histograms of the mean of duration for each AND operation pattern. Solid lines are Gaussian fitted curves for each group of data. All data is obtained through a nanopore with a 3.5 nm diameter nanopore under a voltage of 100 mV.

**Figure 5:**
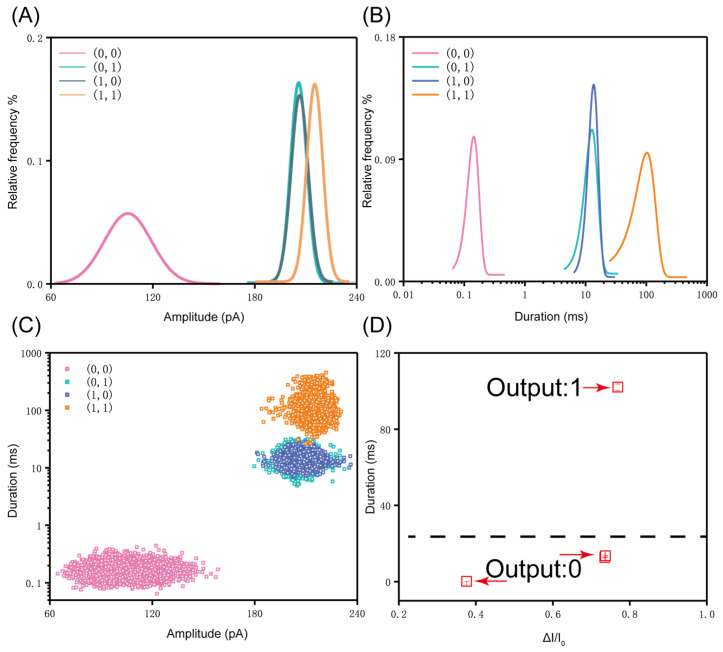
(**A**,**B**) The comparison curve of amplitude and translocation time, the curves are the Gaussian fitting curve obtained from each group of histograms; (**C**) Represents the scatter plot of duration vs. amplitude for four operation patterns under a voltage of 100 mV; (**D**) The dot plot of average duration and normalized amplitude. The figure shows the results of two logical operations (0 and 1).

Analogous to the analysis of amplitude, we also analyzed the translocation time of the four operation patterns, and the histograms of dwell time are shown in Figure 4. The mean duration for (0, 0), (0, 1), (1, 0), (1, 1) is 0.16 ± 0.0004 ms, 13.49 ± 0.107 ms, 13.85 ± 0.051 ms, and 114.04 ± 1.379 ms, respectively. We can observe that the translocations of (0, 1) and (1, 0) show a significant difference from the translocation time of (1, 1). The increase in duration by an order of magnitude from (0, 0) to (0, 1)/(1, 0), and then to (1, 1) is attributed to two factors. On the one hand, the spatial conformation changed after the probe was combined with the DNA marker. On the other hand, the changed molecular conformation exacerbates the interaction between the molecules with the nanopore wall during translocation. Therefore, as compared with the complex of single tetrahedral probe, although the double tetrahedron failed to cause a larger current blockage, the strong interaction with the nanopore wall during translocation obviously improved its translocation time. Similarly, the translocation time of the complex of the single tetrahedral probe and the marker is also significantly higher than the duration of the individual DNA fragment marker. Therefore, we can distinguish (0, 0), (0, 1)/(1, 0), and (1, 1) of operation patterns according to the translocation time. Further normalization of the amplitude reveals a conspicuous difference between (0, 0) and (1, 1). We observe that the deep current blockage exceeds 70% of the threshold level, as shown in Figure 5D. In addition, the scatter plot based on duration and amplitude is shown in Figure 5C. These data graphs help us to improve our experiments and to make our experimental results more convincing.

Finally, we uniformly compare the amplitude and duration obtained from the four groups of logical operations; the results are shown in Figure 5A,B. As presented in Figure 5C, we plotted a scatter plot of four sets of data. Subsequently, we analyzed the average amplitude and mean of duration obtained from each group of DNA logic operations. The specific analysis is shown in Figure 5D. In this analysis, we normalized the average amplitude of each group of data, which better reflected the difference among the four DNA logic operations. According to the analysis of duration and amplitude, the signals of the AND operation (1, 1) can be clearly distinguished from other three operations ((0, 0), (0, 1), (1, 0)), as shown in Figure 5D. This means that when we input two tetrahedral probes at the same time, they can perform the AND operation with the substrate molecule successfully. Combined with the Figures S2 and S3, the logic operation patterns of (0, 1) and (1, 0) are also successfully executed.

## 4. Conclusions

We designed a DNA logic gate (AND gate) based on a tetrahedron probe, including four operation patterns of (0, 0), (0, 1), (1, 0), (1, 1). Different operation patterns correspond to different blocking signals under the measurements of a solid-state nanopore. The double tetrahedron structure corresponding to operation mode (1, 1) caused a long blocking signal when it passed through a nanopore. Combining the translocation time and amplitude of different types of signals, the signals of (1, 1) can be distinguished from the other three operation patterns, which has been confirmed by our analysis of the signals from different operation patterns. In addition, this strategy can be integrated into molecular machines to form a nanopore-based biochemical molecular machine. In the future, due to the ability of parallel computing, DNA computing could solve mathematical problems and could also be applied to study physical and chemical processes of various biomolecules. Therefore, the nanopore platform will become an important tool for DNA computations due to its advantages in the detection process.

## Data Availability

The data presented in this study are available on request from the corresponding author. The data are not publicly available because the research is still ongoing.

## Supplementary Materials

The following are available online at https://www.mdpi.com/1424-8220/21/1/33/s1, Figure S1: Structure diagram of flow cell; Figure S2: Gel electrophoresis of molecules with different structure; Figure S3: Current trace of the tetrahedral probes; Figure S4: The histogram for different tetrahedral probes. Figure S5: Gaussian fitting curves of the histogram for two kinds of tetrahedral probes and DNA logic operation patterns and the scatter plot of amplitude versus duration; Table S1: DNA sequence used in the experiment.
