# Supplementary material for: Recognition of Bimolecular Logic Operation Pattern Based on a Solid-State Nanopore
Source: Sensors (Basel). 2020 Dec 23;21(1):33. doi: 10.3390/s21010033 (PMC7793508; doi:10.3390/s21010033)
Supplement: Supplementary file 1 [file sensors-21-00033-s001.pdf]

Supporting Information for

Identification of biomolecular logic operation pattern based on Solid-state nanopore

Han Yan[†*], Zheng Zhang[†*], Ting Weng[‡], Libo Zhu[†], Pang Zhang[‡], Deqiang Wang[‡], Quanjun Liu[*†]

[†]State Key Laboratory of Bioelectronics, School of Biological Science and Medical Engineering, Southeast University, No. 2, Sipailou, Nanjing 210096, P. R. China.

[‡]Chongqing Institute of Green and Intelligent Technology, Chinese Academy of Sciences, Chongqing, 400714, P. R. China.

[*]E-mail: lqj@seu.edu.cn

Table of Contents

Figure S1

Structure diagram of flow cell.

Figure S2

Gel electrophoresis of molecules with different structures.

Figure S3

Current trace of the tetrahedral probes.

Figure S4

The histogram for different tetrahedral probes.

Figure S5

Gaussian fitting curves of the histogram for two kinds of tetrahedral probes and DNA logic operation patterns and the scatter plot of amplitude versus duration.

Table S1

DNA sequence used in the experiment

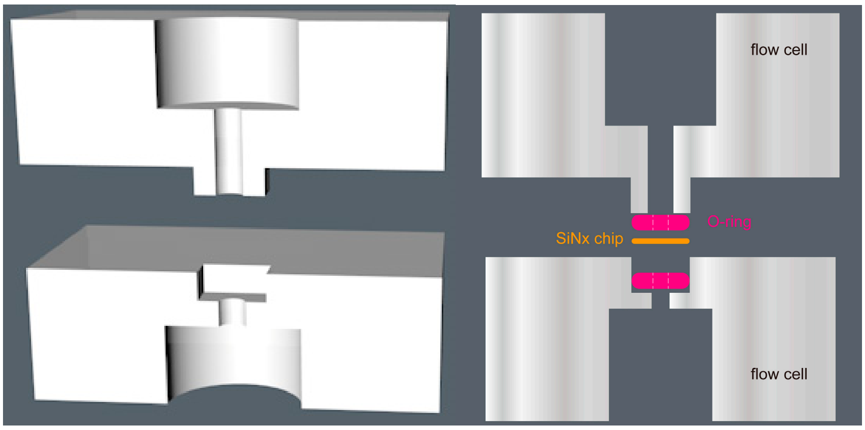

Figure S1. Structure diagram of flow cell. The picture shows the two chambers of the flow cell, the sealing rubber ring and how to install the nanopore chip.

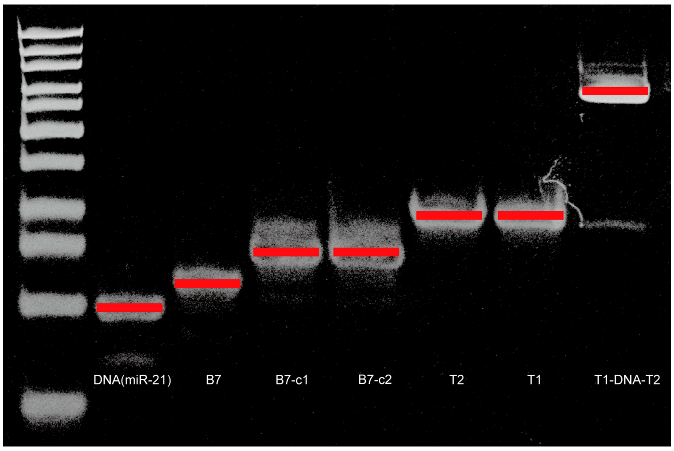

Figure S2. Electrophoretogram of different biomolecules. Different bands represent different biomolecules and the corresponding DNA tetrahedral structure.

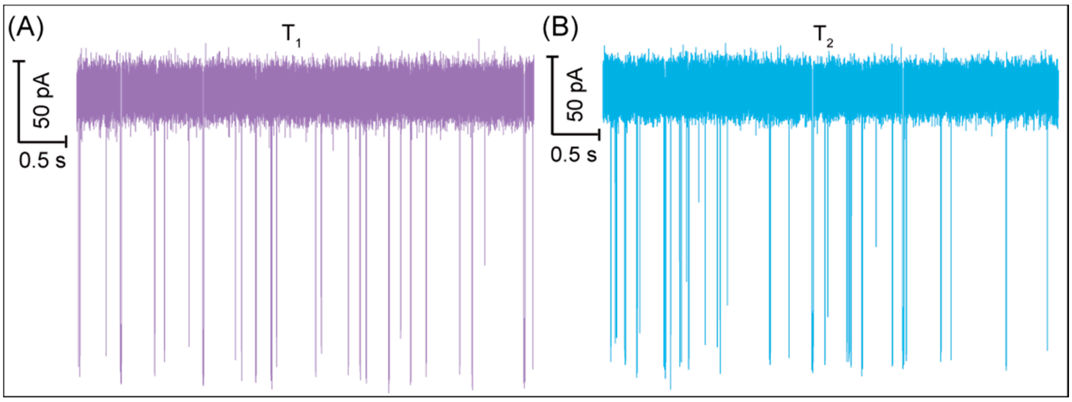

Figure S3. (A) and (B) Current trace of the tetrahedral probes, respectively. T1 tetrahedral probe is composed of A7, B7-C1, C7 and D7, while T2 tetrahedral probe is composed of A7, B7-C2, C7 and D7.

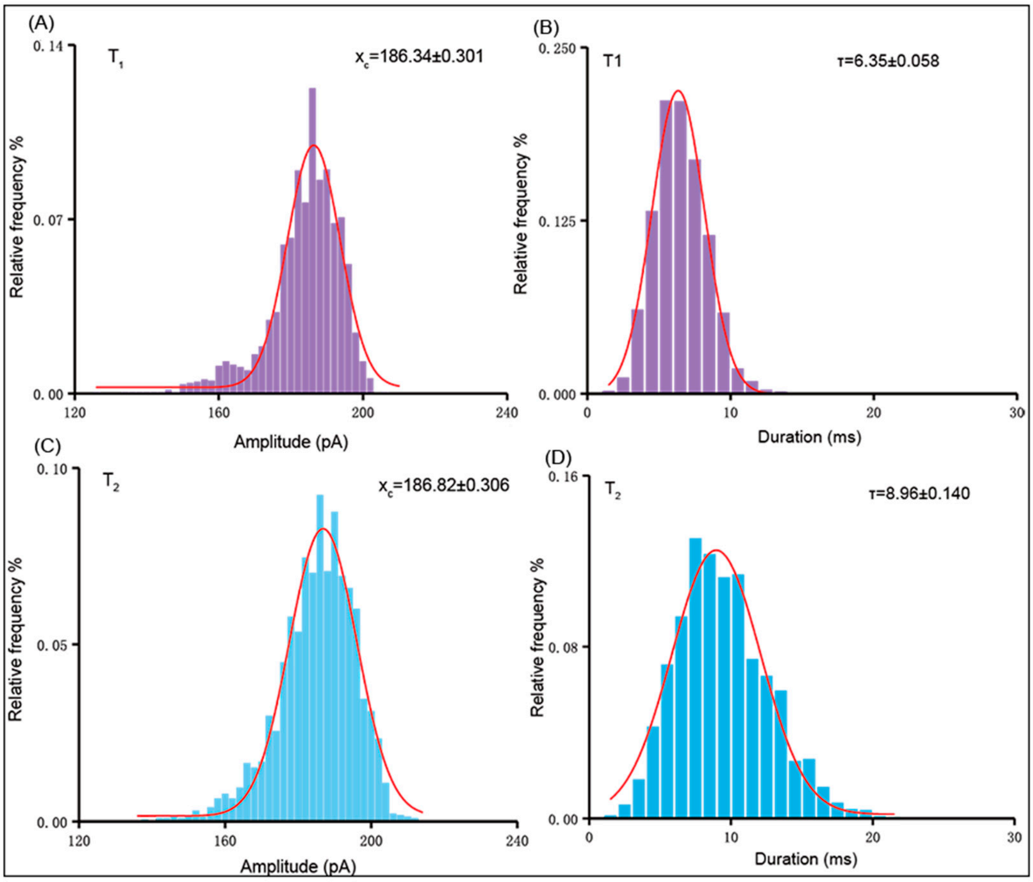

Figure S4. (A)-(D) The histogram of the amplitude and duration from different tetrahedral probes (T_1 and T_2), respectively. The solid curves in the figure are the Gaussian fitting curve.

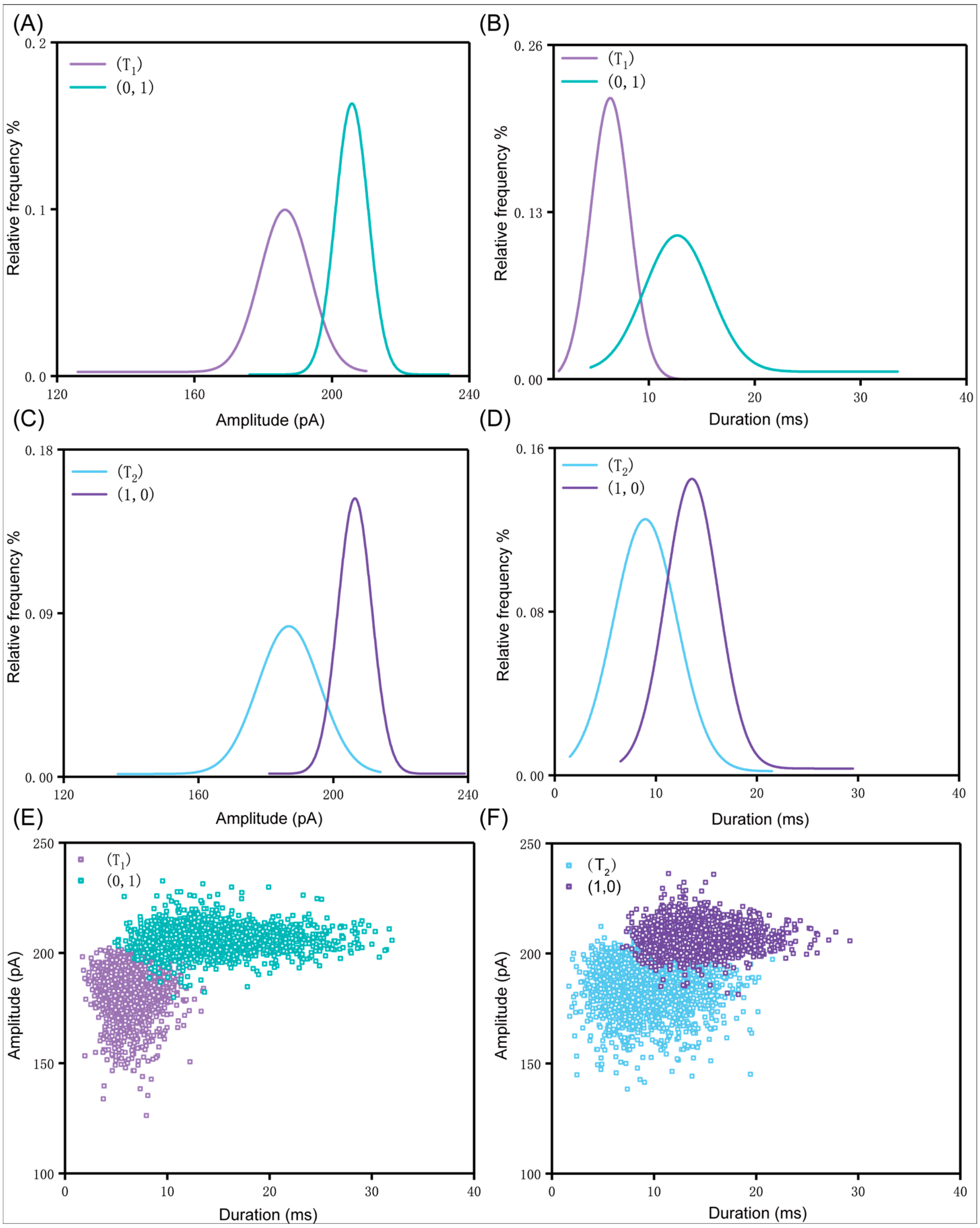

Figure S5. (A)-(D) Gaussian fitting curves of the histogram for two kinds of tetrahedral probes (T_1 and T_2) and DNA logic operation patterns of (0,1) and (1,0) at +100 mv voltage, respectively. (E)-(F) The scatter plot of amplitude versus duration for two probes and two operation patterns.

Table S1. DNA sequence used in the experiment. Letters of the same color represent complementary parts.

DNA	Base sequence
DNA(miR-21)	TAGCTTATCAGACTGATGTTGA
DNA fragment	TAGCTTTGTTGA
A7	GAGCGTTAGCCACACACACAGTC
C7	CGCCTAAACAAGTGGAGACTGTG
D7	AACGCTCACCACTTG AACACCTC
B7-C1	TTAGGCGAGTGTGGCAGAGGTGTTTCAACA
B7-C2	AAGCTATTTAGGCGAGTGTGGCAGAGGTGT
